# Intestinal microRNAs and bacterial taxa in juvenile mice are associated, modifiable by allochthonous lactobacilli, and affect postnatal maturation

**DOI:** 10.1128/msystems.00431-23

**Published:** 2023-07-18

**Authors:** Amel Taibi, Tomas Tokar, Julien Tremblay, Giorgio Gargari, Catherine J. Streutker, Bo Li, Agostino Pierro, Simone Guglielmetti, Thomas A. Tompkins, Igor Jurisica, Elena M. Comelli

**Affiliations:** 1 Department of Nutritional Sciences, Faculty of Medicine, University of Toronto, Toronto, Ontario, Canada; 2 Krembil Research Institute, University Health Network, Toronto, Ontario, Canada; 3 Energy, Mining and Environment, National Research Council Canada, Montréal, Quebec, Canada; 4 Department of Food Environmental and Nutritional Sciences (DeFENS), University of Milan, Milan, Italy; 5 Department of Laboratory Medicine and Pathobiology, Unity Health Toronto: St. Michael’s Hospital, Toronto, Ontario, Canada; 6 Division of General and Thoracic Surgery, Physiology and Experimental Medicine Program, The Hospital for Sick Children, Toronto, Ontario, Canada; 7 Lallemand Bio-Ingredients, Montréal, Quebec, Canada; 8 Department of Medical Biophysics, University of Toronto, Toronto, Ontario, Canada; 9 Department of Computer Science, University of Toronto, Toronto, Ontario, Canada; 10 Osteoarthritis Research Program, Division of Orthopedic Surgery, Schroeder Arthritis Institute, University Health Network, Toronto, Ontario, Canada; 11 Joannah and Brian Lawson Centre for Child Nutrition, Faculty of Medicine, University of Toronto, Toronto, Ontario, Canada; University of California, San Diego, La Jolla, California, USA

**Keywords:** microRNAs, microbiota, postnatal development, intestinal maturation, probiotics

## Abstract

The interplay between the intestinal microbiota and host is critical to intestinal ontogeny and homeostasis. MicroRNAs (miRNAs) may be an underlying link. Intestinal miRNAs are microbiota-dependent and, when shed in the lumen, affect resident microorganisms. Yet, longitudinal relationships between intestinal tissue miRNAs, luminal miRNAs, and luminal microorganisms have not been elucidated, especially in early life. Here, we investigated the postnatal cecal miRNA and microbiota populations, their relationship, and their impact on intestinal maturation in specific pathogen-free mice; we also assessed if they can be modified by intervention with allochthonous probiotic lactobacilli. We report that cecal and cecal content miRNA and microbiota signatures are temporally regulated, correlated, and modifiable by probiotics with implications for intestinal maturation. These findings help understand causal relationships within the gut ecosystem and provide a basis for preventing and managing their alterations in diseases throughout life.

**IMPORTANCE:**

The gut microbiota affects intestinal microRNA (miRNA) signatures and is modified by host-derived luminal miRNA. This suggests the existence of close miRNA-microbiota relationships that are critical to intestinal homeostasis. However, an integrative analysis of these relationships and their evolution during intestinal postnatal maturation is lacking. We provide a system-level longitudinal analysis of miRNA-microbiota networks in the intestine of mice at the weaning transition, including tissue and luminal miRNA and luminal microbiota. To address causality and move toward translational applications, we used allochthonous probiotic lactobacilli to modify these longitudinal relationships and showed that they are critical for intestinal maturation in early life. These findings contribute to understand mechanisms that underlie the maturation of the intestinal ecosystem and suggest that interventions aiming at maintaining, or restoring, homeostasis cannot prescind from considering relationships among its components.

## INTRODUCTION

The gut microbiota is a determinant of health (1), which relies on its crosstalk with the host. This interaction is established in early life, but underlying mechanisms are incompletely understood. Systems biology approaches unveiled the importance of the intestinal microbiota acting as an environmental factor that drives host gene expression by signaling to genetically pre-disposed cells (2). Microbiota-dependent epigenetic regulation, though, was shown not to be critical, at least at the methylome level (3). However, microRNAs (miRNAs) as an epigenetic dimension remain under-investigated. Intestinal miRNAs regulate development and maturation (4, 5), and cross-sectional studies in adult mice demonstrated that their expression is microbiota-dependent (6 - 10). Intestinal miRNAs are also released into the lumen (luminal or content miRNAs) (11, 12), where they impact the gut microbiota (11, 13) and can be ultimately recovered in the feces (fecal miRNAs). Fecal miRNAs have been proposed as diagnostic markers of disease (14). We hypothesized the existence of regulatory networks between intestinal tissue miRNAs and luminal miRNAs that connect with microbial signatures, potentially underlined by causal relationships. These are likely critical during postnatal maturation, but the longitudinal remodeling of these interactions has not been investigated. Moreover, altered microbiota-host relationships in early life are a known predictor of later disease (15) and may affect lifelong health; though, their inherent plasticity makes them susceptible to environmental stimuli and interventions, which may be clinically relevant. Thus, we investigated the re-shaping of the cecal and cecal content miRNA signatures and their associations with the maturing microbiota before, at, and after the weaning transition in mice. To contribute to the understanding of causality, we then assessed how these longitudinal relationships are altered in response to an intervention with allochthonous probiotic lactobacilli. We used a mix of two lactobacilli strains correctly defined as probiotics according to WHO and expert consensus guidelines (16, 17), namely Lacidofil, which holds approved health claims for children and adults from Health Canada (18) and Brazil (19). This mixture contains two strains isolated from dairy starters and was previously shown to sustain intestinal homeostasis in children and adults [reviewed in reference (20)]. Lactobacilli were previously shown to induce miRNA responses (21, 22). In this study, we found that the intestinal miRNAs (miRNome) and microbiota co-evolve during postnatal maturation, with implications for intestinal maturation via increased cell proliferation, which is modifiable and enhanced by probiotic lactobacilli.

## RESULTS

### Study design, dam and litter characteristics, and recovery of probiotic strains

**Fig 1:**
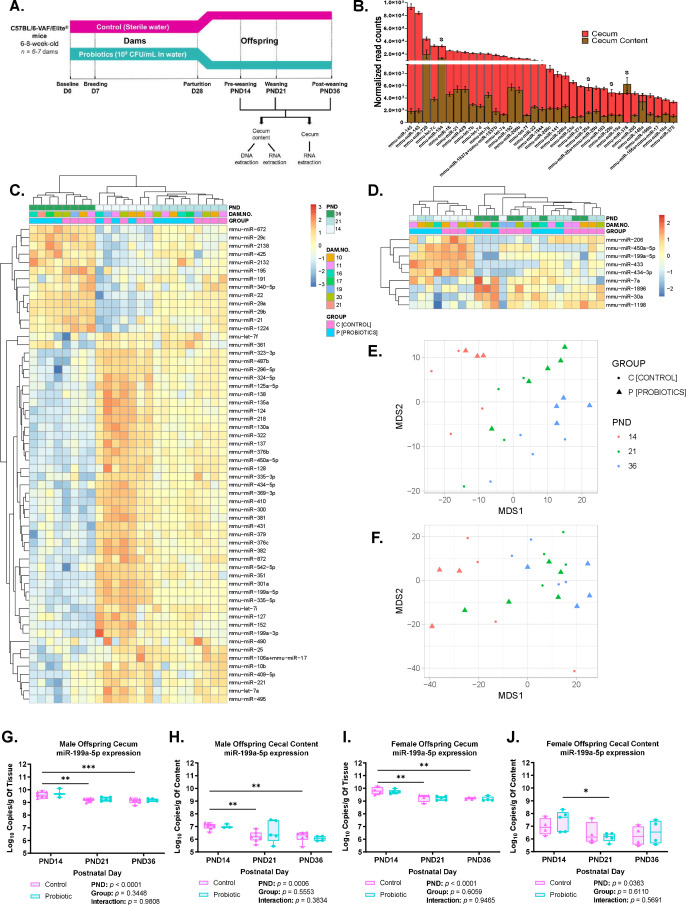
Cecal and cecal content microRNA signatures depend on postnatal age and partially overlap. (**A**) Experimental approach as described in Materials and Methods. (**B**) Bar plot of the most abundant 35 miRNAs shared between cecum and its content (mean ± SEM of normalized read counts; *n* = 24 male offspring for each of cecum and its content). *S* indicates significantly positively correlated miRNAs between cecum and its content. See Table S1 for complete list. (**C and D**) Heatmap showing unsupervised hierarchical clustering of significantly differentially expressed PND-associated miRNAs in cecum (**C**) (FDR-adjusted *P*-value < 0.01) and its content (**D**) (FDR-adjusted *P*-value < 0.15). Significance assessed by regression analysis followed by ANOVA. The colors indicate the intensity of z-scored log_2_ normalized miRNA counts (red, high expression; blue, low expression). See Tables S2 and S3 for the cecum PND-associated predicted miRNA-gene targets and enrichment analysis, respectively. (**E and F**) Multidimensional scaling (MDS) plots of miRNA normalized expression profile of cecum (**E**) and its content (**F**) visualizing sample similarities by PND and group (*n* = 3–5 male offspring/PND/group for each of cecum and its content). (**G–J**) ddPCR absolute quantification of miR-199a-5p in cecum (**G and I**) and its content (**H and I**) in male (**G and H**) and littermate female offspring (**I and J**). Data presented as mean ± SEM of log_10_ copies/g of cecum or content (*n* = 3–7 offspring/sex/PND/group) (two-way ANOVA and FDR-adjusted *P*-value; **P < 0.01, ***P < 0.001, ****P < 0.0001).

C57BL/6-VAF/Elite mice were randomized to the control or probiotics group and mated 1 week later. Littermate male and female pups were euthanized at postnatal days (PNDs) 14, 21 (weaning), and 36, and cecum and its content were collected (Fig. 1A). There was no effect of maternal probiotic administration on dams, litter, and offspring body weight growth curves, litter size, and sex ratio (Fig. S1A through G). Probiotic strains were detected in the cecal content of pups exposed to probiotics but not in the control group (Fig. S4C through F).

### Cecal and cecal content microRNA signatures depend on postnatal age and partially overlap

To investigate the murine developmental miRNA signatures in the cecum, paired cecal and cecal content samples before, at, and after the weaning transition were used.

Bioanalyzer electropherograms of total RNA samples showed different peaks of RNA species, including of the miRNA size (Fig. S2A and 2B) as previously identified in adult mouse feces (11). Thus, juvenile mice cecal content contains miRNAs. Cecal and cecal content miRNAs were profiled using NanoString nCounter Technology (23).

A total of 451 and 505 miRNAs were expressed in the cecum and its content, respectively, with 425 miRNAs shared between them (Table S1) and (Fig. 1B). Pearson correlations identified 28 positively and 1 negatively correlated miRNAs between paired samples (Table S1).

In cecum, we identified 58 differentially expressed PND-associated miRNAs (Fig. 1C); 13 and 45 miRNAs were up- and down-regulated, respectively, at PND36 vs PND14 and PND21 (*β*_1_ = 0.0091 to 0.1202; *β*_1_ = −0.2475 to −0.0129). Hierarchical clustering showed PND-associated grouping (Fig. 1C). This longitudinal variation was confirmed in the multidimensional scaling (MDS) plot of the normalized miRNA expression (Fig. 1E). Using miRWalk2.0, 9,400 PND-associated miRNA-gene targets were predicted (Table S2). Gene Ontology (GO) enrichment analysis identified 785 enriched GO annotations across GO biological processes (GObp), molecular functions (GOmf), and cellular components (GOcc), in particular, development- and proliferation-related processes (Table S3).

In cecal content, nine PND-associated miRNAs were differentially expressed (Fig. 1D), with clustering observed primarily at and after the weaning transition (Fig. 1D and F). Four of these were upregulated (*β*_1_ = 0.0485 to 0.1717) and five were downregulated (*β*_1_ = −0.2086 to −0.0869) at PND36 and PND21 vs PND14. MiR-450a-5p and miR-199a-5p were downregulated and shared between the cecum and cecum content signatures. MiR-450a-5p was the only miRNA significantly associated with dam and thus excluded from further analyses. MiR-199a-5p ranked among the 30 most highly expressed miRNAs in the cecum and one of the most significantly positively correlated miRNAs between the cecum and its content (Table S1). MiR-199a-5p downregulation in paired cecum and content (Fig. 1G through J) and its positive correlation between the two (Pearson *r* = 0.5785, *P*-value < 0.0001; data not shown) were confirmed by Droplet Digital PCR (ddPCR) in both male and female offspring. Interestingly, miR-199a-5p NanoString log_2_ normalized counts were significantly positively correlated with miRNA copy number quantified by ddPCR (log_2_ copies) in both cecum (Pearson *r* = 0.7578, *P*-value < 0.0001) and its content (Pearson *r* = 0.7246, *P*-value < 0.0001) (Fig. S2C and D). These findings provide a rationale for miR-199a-5p as a potential molecular marker for postnatal development.

### Probiotics affect the maturation of the cecum and cecum content microRNA signatures and increase proliferation

**Fig 2:**
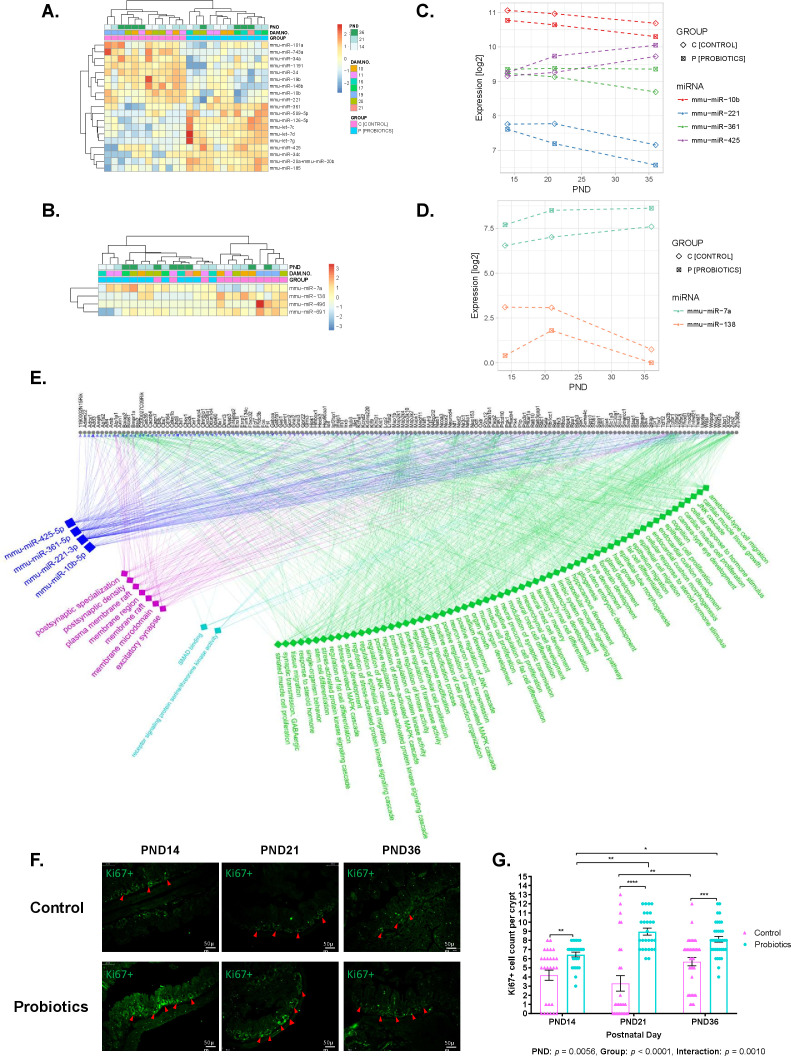
Probiotics affect the maturation of the cecum and cecum content microRNA signatures and increase proliferation. (**A and B**) Heatmap showing unsupervised hierarchical clustering of group-associated, significantly deregulated miRNAs in cecum (A, FDR-adjusted *P*-value < 0.01) and its content (B, FDR-adjusted *P*-value < 0.25) (*n* = 3–5 offspring/sex/PND). Significance assessed by regression analysis followed by ANOVA. The colors indicate the intensity of z-scored log_2_ normalized miRNA counts (red, high expression; blue, low expression). See Tables S2 and S3 for the group-associated predicted miRNA-gene targets and enrichment analysis, respectively. (**C and D**) Panel plots showing significantly differentially expressed PND- and group-associated miRNAs in cecum (C, FDR-adjusted *P*-value < 0.01) and its content (D, FDR-adjusted *P*-value < 0.25). Significance assessed by regression analysis followed by ANOVA. Panel plots show PND on x-axis and log_2_ normalized miRNA counts on y-axis. See Tables S2 and S3 for the cecum PND- and group-associated predicted miRNA-gene targets and enrichment analysis, respectively. (**E**) Putative regulatory network of the four PND- and group-associated miRNAs in the cecum (blue squares) and their predicted gene targets (gray circles) enriched in Gene Ontology (GO) biological processes (GObp) (green diamonds), molecular functions (GOmf) (teal diamonds), and cellular components (GOcc) (purple diamonds) (FDR-adjusted *P*-value < 0.01). Edges are represented by blue for miRNA-gene targets and depicted by green, teal, and purple for genes enriched in GObp, GOmf, and GOcc, respectively. (**F**) Ki67 immunofluorescence (green) of cecal epithelial cell proliferation in control and probiotics groups and (**G**) mean ± SEM of Ki67+ cell count/crypt (*n* = 9 replicates for 3–4 offspring/PND/group; two-way ANOVA and Bonferroni-adjusted *P-value*; *P < 0.05, **P < 0.01, *** *P* < 0.001, ****P < 0.0001. See **Fig. S3A through D** for H&E and AB/PAS staining and scoring of cecal tissue.

The PND-associated miRNAs further sub-clustered by group (probiotics vs control) at PND21 and PND36 (Fig. 1C). To determine whether the maturing cecal miRNA signature responds to, and thus can be modified by environmental cues, we analyzed the miRNA profiles for group as a predictor. In cecum, 19 differentially expressed group-associated miRNAs were identified (Fig. 2A), 10 miRNAs were upregulated (*β*_2_ = 0.2371 to 4.1619) and 9 were downregulated (*β*_2_ = −3.8736 to −0.2362) in the probiotics vs control group. These miRNAs clustered perfectly based on group. Using miRWalk2.0, a total of 4,591 group-associated miRNA-gene targets were predicted (Table S2) and 189 enriched GO annotations were identified (Table S3). In the cecal content, four differentially expressed group-associated miRNAs were identified (Fig. 2B), one miRNA was upregulated (*β*_2_ = 1.9580) and three were downregulated (*β*_2_ = −1.7243 to −0.7022) in probiotics vs control group. These miRNAs clustered primarily based on group, suggesting that probiotics affect the intestinal miRNA signatures.

To determine whether probiotic supplementation affects cecal and cecal content miRNA signatures after birth, the miRNA data were analyzed using PND and group as predictors. Four differentially expressed PND- and group-associated miRNAs were identified in the cecum (Fig. 2C), two miRNAs were upregulated (*β*_1_ = −0.0170 to 0.0318, *β*_2_ = 0.2371 to 0.2665) and two were downregulated (*β*_1_ = −0.0358 to −0.0213, *β*_2_ = −0.4334 to −0.3517) in probiotics vs control group. These had 465 predicted gene targets (Table S2) and were enriched in 78 GO annotations (Table S3).

In cecal content, two differentially expressed PND- and group-associated miRNAs were identified (Fig. 2D); one miRNA was upregulated (*β*_1_ = 0.0485, *β*_2_ = 1.9580) and one miRNA was downregulated (*β*_1_ = −0.0781, *β*_2_ = −1.3535) developmentally in the probiotics vs control group.

To understand if intestinal miRNA signatures in the probiotic group at the earlier time-points (PND14 and 21) resemble those of the control group at the later time-points (PND21 and 36), paired comparisons of cecal and cecal contents data were conducted. In the cecum, 34 and 77 miRNAs were significantly differently expressed in probiotic group at pre-weaning (PND14) compared to the control group at PND21 (weaning) and PND36 (post-weaning), respectively (FDR-adjusted *P*-value < 0.05) (Fig. S2E and F and Table S4). A total of 11 (out of 34) and 34 (out of 77) were identified as PND-associated miRNAs, including miR-199-5p (Fig. S2E and F; Table S4). Similarly, mice exposed to probiotics at weaning (PND21) exhibited significant changes in the expression level of 54 miRNAs when compared to control-treated mice after weaning (PND36) (Fig. S2G and Table S4). Of these, 27 and 7 miRNAs were identified as PND- and group-associated miRNAs, respectively. As expected, probiotic-associated miRNAs were found to discriminate between the groups after weaning only (probiotic at PND21 vs control at PND36; Table S4).

In the cecum content, 11 miRNAs were found to be significantly differentially expressed in pre-weaned probiotic-treated pups (PND14), compared to the ones from the control group at weaning (PND21) (Fig. S2H). None of them was found to be PND- nor group-associated miRNA in our previous analysis. MiRNA expression levels of probiotic-treated mice at PND14 (pre-weaning) and PND21 (weaning) were comparable to the ones of control-treated mice at PND36 (post-waning) (Fig. S2I and J).

The four PND- and group-associated miRNAs in cecum (Fig. 2C) and their gene targets enriched in GO annotations across 69 GObp, 2 GOmf, and 7 GOcc terms (Table S3) were used to construct a regulatory network (Fig. 2E). This highlighted several cell differentiation- and proliferation-related GO terms enriched among our miRNA-mRNA pairs. Ki67 proliferation marker protein staining confirmed a significant PND × group interaction with Ki67+ cell count/crypt being significantly higher in the probiotics vs control group at each PND (Fig. 2F and G). This is aligned with increased crypt depth and goblet cell numbers in the probiotics group (Fig. S3A through D), suggesting that probiotics increase epithelial cell proliferation via miRNA.

### Probiotics modify the maturation of the cecal microbiota

Previous studies reported that intestinal proliferation genes depend on the composition of the microbiota (8 - 10). To assess whether maternally administered probiotics affect the offspring cecal microbiota postnatally, we ran 16S rRNA gene sequencing analysis of cecal content DNA.

**Fig 3:**
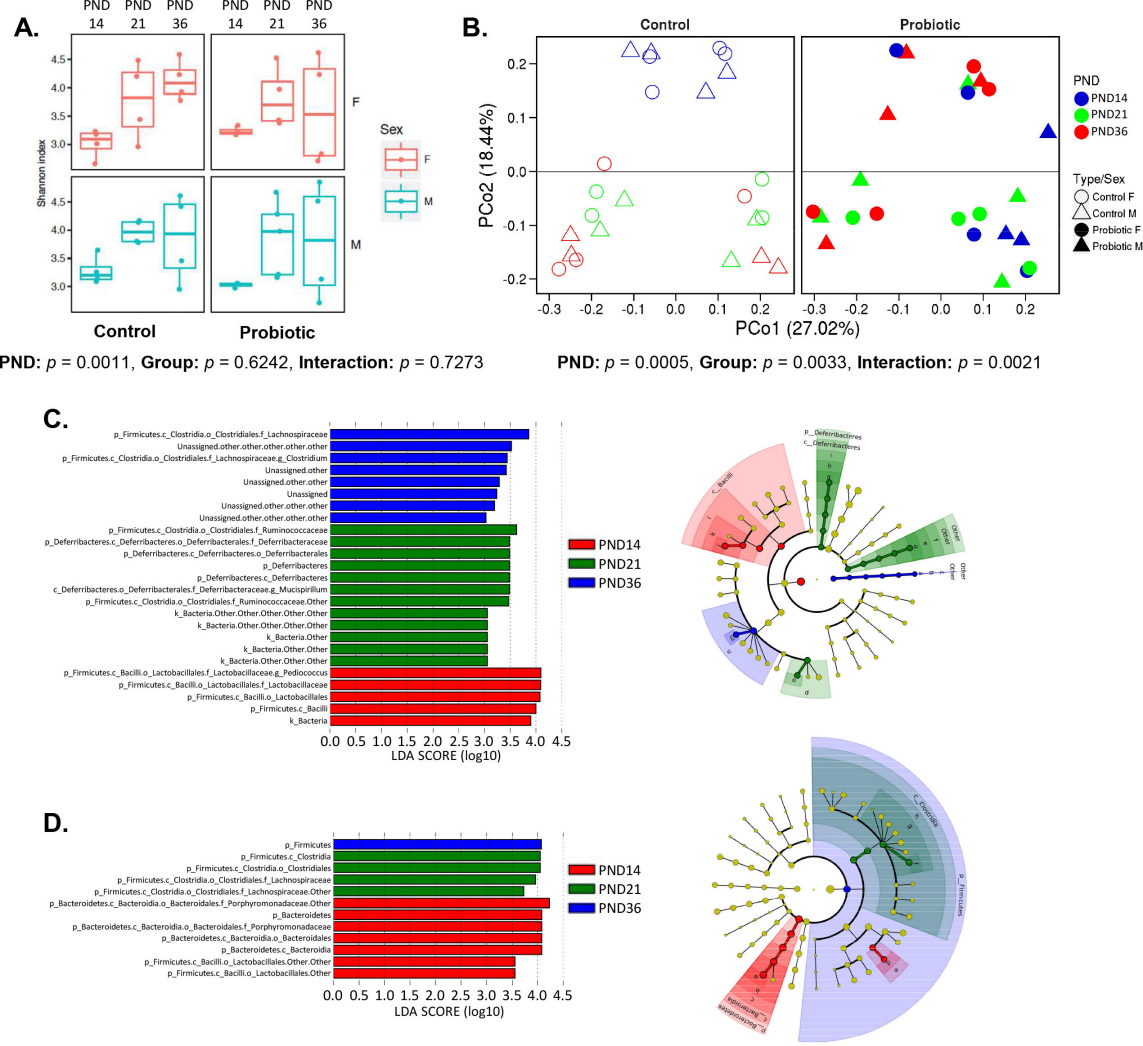
Probiotics modify the maturation of the cecal microbiota. (**A**) Alpha diversity measurement of the Shannon index where box plots represent mean ± SEM; each dot represents an individual offspring (*n* = 7–10 offspring/PND/group). A significant main PND effect was observed (two-way ANOVA and Tukey-adjusted *P-value*, PND: *P*-value = 0.0011). (**B**) Beta diversity depicted by principal coordinates analysis (PCoA) of the Bray–Curtis dissimilarity matrix (plots separated for clarity); each dot represents an individual offspring (*n* = 7–10 offspring/PND/group). A significant PND x group interaction was observed (ADONIS permutation-based function and *P*-value = 0.0021). (**C and D**) LEfSe analysis showing age-discriminating taxa in the control (**C**) and probiotic (**D**) groups (*n* = 7–10 offspring/PND/group). The left histograms show the log_10_ linear discriminant analysis (LDA) scores computed for the relative abundance of taxa differentially abundant between the postnatal days in the control (**C**) and probiotic (**D**) groups (significance at *P*-value < 0.05 and absolute LDA (log_10_) scores > 2.0). The cladograms on the right show the differences in the relative abundance of taxa at five levels (**L2–L6**) in control (**C**) and probiotic (**D**), according to the LEfSe analysis (LDA threshold of 2.0, FDR-adjusted *P*-value < 0.05). See also Fig. S4G–L and S5A–C for differentially abundant OTUs identified using DESeq2 method.

After filtration and quality controls, a range of 81,793 to 270,781 sequences were generated and clustered into 2,464 operational taxonomic units (OTUs). Alpha-diversity (Shannon index) increased with age (Fig. 3A) with no difference between groups, indicating that postnatal age, but not probiotics, affects microbial diversity and richness. This was aligned with total bacterial counts increasing with time but no effect of probiotics (Fig. S4A and B). Conversely, treatment was more important than age in distinguishing microbiota structure (Fig. 3B). The Bray–Curtis dissimilarity matrix revealed a significant age-dependent distinction with PND14 separating from PND21 and PND36 in control mice (Fig. 3B). This is in line with PND-associated miRNA signature in the content, where pre-weaning samples were clustered separately from PND21 and PND36.

On the other hand, age-dependent segregation of mouse cecal microbiota structures was drastically reduced in the probiotics group, suggesting a contribution of probiotic lactobacilli to microbiota maturation (Fig. 3B). These findings were confirmed by the taxonomical data, where a larger number of taxa were found to discriminate microbiota samples longitudinally in control vs probiotics group. In particular, the genera *Pediococcus* (*Lactobacillaceae* family), *Mucispirillum* (*Deferribacteres* phylum), and *Clostridium* (*Lachnospiraceae* family) were identified by both LEfSe and DESeq2 analyses as significantly enriched at PND14, PND21, and PND36, respectively (Fig. 3C; Fig. S4G through I), with limited differences in the probiotic mice (Fig. 3D; Fig. S4J through L).

**Fig 4:**
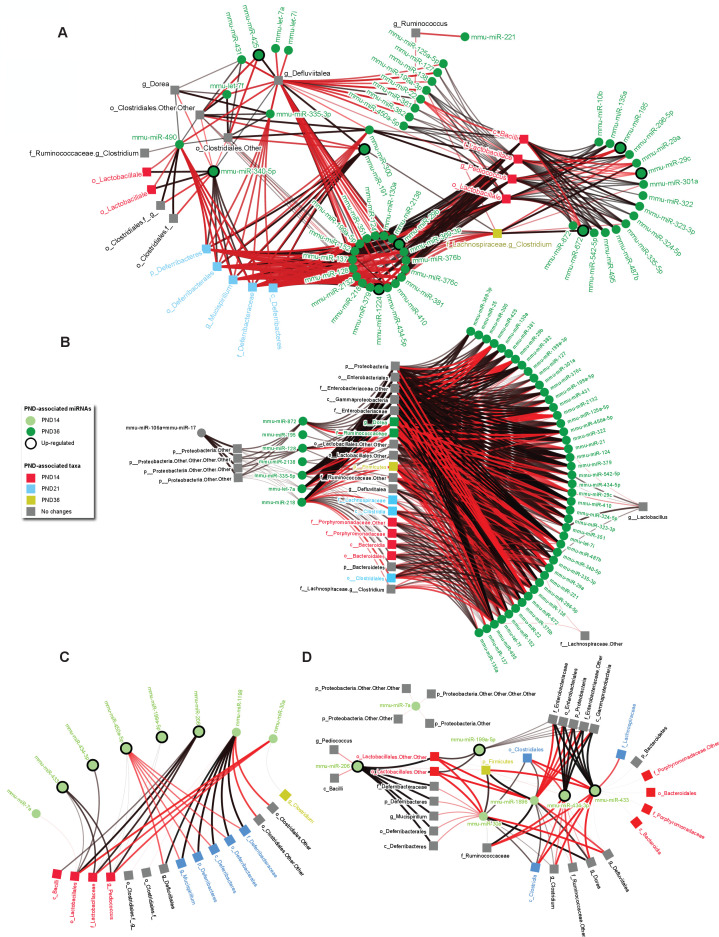
Maturing microRNA and microbiota signatures are correlated, and probiotics modify their relationship. (**A and B**) Networks showing the significant correlations between PND-associated miRNAs and bacterial taxa in the cecum of the control (**A**) and probiotic (**B**) groups (*T* ≥ 0.44, *P*-value < 0.05). (**C and D**) Networks showing the significant correlations between PND-associated miRNAs and bacterial taxa in the cecal content of the control (**C**) and probiotic (**D**) groups. (**A-D**) Node colors refer to the age-associated miRNA and/or taxa significant changes. Red and black edges represent the negative and positive significant correlations, respectively (*T* ≥ 0.44, *P*-value < 0.05). The networks were visualized using NAViGaTOR 3.0 (24).

In line with this, the aforementioned taxa were also identified as significantly differently abundant in the pre-weaned probiotic pups (PND14), compared to the ones from the control groups at later time-points (PND21 and PND36) (Fig. S5A and B). The microbiota in the probiotic group at weaning (PND21) was similar to that in the control group at PND36 (Fig. S5C).

### Maturing microRNA and microbiota signatures are correlated, and probiotics modify their relationship

To investigate the relationship between cecal miRNAs and microbiota, we performed a pairwise Kendall correlation analysis of the cecal content bacterial taxa (taxonomical levels from phylum to genus) and miRNAs expressed in the cecum and its content. 231 and 194 miRNAs in the control group and 192 and 208 miRNAs in the probiotics group were correlated with at least one taxon in the cecum and its content, respectively (Table S5).

Next, we dissected the PND-dependency within these correlations for either miRNA or taxa. For cecal miRNA, 54 and 52 of these taxon-correlated miRNAs were previously (Fig. 1C) identified as PND-associated in the control and probiotics groups, respectively (Fig. 4 and B), with 11 miRNA-taxon pairs being common in the two groups (Fig. S6A). In the cecum content, eight and seven miRNAs were previously (Fig. 1D) identified as PND-associated in the control and probiotics groups, respectively (Fig. 4C and D), with three miRNA-taxon pairs being content-specific (Fig. S6B). This shows both shared and site (cecum vs content)-specific miRNA-taxon pairs. The effect of the probiotics is shown in correlation networks visualizing pairwise miRNA-taxon associations (Fig. 4B and D). There was a higher connectivity for pairs in the probiotics group vs control, in both cecum and its content (Fig. 4A through D), with 550 (cecum) and 84 (cecum content) miRNA-taxon pairs identified in the probiotics group compared to 366 (cecum) and 33 (cecum content) in the control group. In particular, miR-199a-5p-taxon correlation pairs were group-specific.

For taxa, at least half of the significant correlations in the control group in both cecum and content involved PND-associated taxa that were found to discriminate between the control and the probiotics group, including *Pediococcus, Mucispirillum,* and *Clostridium* genera (Fig. 3C and D; Fig. 4A and C). Interestingly, most of these PND-associated correlations were modified by probiotics (Fig. S6C and D).

Together, these data suggest that the gut microbiota composition is associated with miRNA expression and specifically with selected miRNAs that are developmentally regulated and modified in response to probiotics supplementation.

## DISCUSSION

Here, we report that cecal and cecal content miRNA signatures are postnatally regulated in early life and that a relationship exists between them and with the luminal microbiota. Age-dependent miRNAs, including those that are significantly correlated with microbial taxa, target cell growth, epithelial cell proliferation and differentiation, and tissue development. This suggests the existence of a tripartite interaction among tissue and luminal miRNAs and luminal microbiota which is critical for intestinal maturation, providing a system-level perspective on epigenetic regulation of this postnatal process. The importance of microbial cues in this context is confirmed by our finding that allochthonous microorganisms modify the relationships. Specifically, probiotic lactobacilli enhance miRNA-taxon pair connectivity, which is accompanied by enhanced epithelial proliferation and age-independent modulation of cecal microbiota community structure. This is important because the microbiota was previously found to only minimally affect intestinal gene expression epigenetically via DNA methylation processes (3). Our findings show that the miRNA dimension is, on the other hand, cardinal. This offers a possible explanation for personalized microbial responses to interventions, especially in early life, when not only microbial, but also as shown here, miRNA signatures, are plastic and modifiable. Interestingly, we found that the expression of age-dependent miR-199a-5p in the lumen and tissue is correlated, suggesting a potential use of this miRNA as a biomarker of microbiota maturation. Our data also suggest that studies cannot prescind from comprehensive analyses, since multiple miRNAs work collaboratively and together with the microbial community.

The modifying effects of the probiotics on the infant intestinal miRNA-microbiota relationships is remarkable, primarily since it was observed in healthy animals and since it highlights the vast potential of this intervention.

The PND-associated miRNA*-*gene targets were previously identified as microbiota responsive (3, 7), including a subset found to be developmentally regulated in mice (3). The fact that these genes are likely regulated via miRNAs that respond to the microbiota opens the horizon for interventions that target the microbiota for fine-tuning host gene expression. In addition, the probiotic used here modified the maturation of the cecal microbiota making the microbiota of probiotic treated mice at PND14 undistinguishable from that of control mice at PND36. These findings provide a rationale for probiotics administration to infants at risk of developmental delays, including malnourished infants and those born preterm who have a slower and/or disturbed maturation of their intestinal microbiota (25, 26). The use of a substantiated probiotic supports translatability of our findings.

This withstanding, broad implication must be considered. Probiotics increased miRNA-taxon interconnectivity and reversed the correlations of selected age-discriminatory taxa with PND-associated miRNAs (Fig. S6C through D). Interestingly, microbiota modulation was not necessary for these probiotic effects, suggesting a direct impact of the lactobacilli administered on the intestine. While the end result appears to be beneficial, long-term consequences need to be investigated. In addition, a diseased intestine may or may not be able to respond to probiotics via miRNA in a similar manner as shown here. Finally, inanimate microorganisms or their metabolites may also elicit miRNA responses (27), suggesting that para- or post-biotics have the potential for future investigation and in clinical applications.

In summary, our study outlines the effect of postnatal development on intestinal and luminal miRNA signatures. It shows that the epigenetic process is associated to the developmental changes of the intestinal microbial ecosystem, dependent on microbial cues. Our data show the impact of probiotic lactobacilli in shaping the bidirectional association between miRNA-microbiota during postnatal maturation with potential implications for healthy growth. Given that the probiotic effects are strain-specific, future studies addressing the impacts of the individual strains *L. rhamnosus* R0011 and *L. helveticus* R0052 should help in identifying strain-specific miRNAs-microbes networks.

## MATERIALS AND METHODS

### *In vivo* animal study

Thirteen female and seven male C57BL/6-VAF/Elite mice were purchased at 6–8 weeks of age from Charles River Laboratories (Saint-Constant, QC, Canada) and housed in sterile conditions throughout the study under a 14:10 light:dark cycle and at room temperature. Mice received sterile water and Teklad Irradiated Global 18% Protein Rodent Diet (Envigo, Madison, WI, USA) *ad libitum*. After 1-week acclimatization, and on Day 0 of the study, female mice were randomized into two groups to continue receiving sterile water (control group, *n* = 7) or water supplemented with 10^9^ colony-forming units (CFU) of a mixture of *Lacticaseibacillus rhamnosus* R0011 (95%) and *Lactobacillus helveticus* R0052 (5%) per mL (Probiotics group, *n* = 6) (Fig. 1A). Water was changed every 2 days. On Day 7, mice were bred harem-style (M:F = 1:2–3); following confirmation of pregnancy, the dams were housed individually. After parturition, litters were followed longitudinally. Dams (postnatal day, PND, 21) and one male and one female pup/litter (PND 14, 21 [weaning] and 36) were sacrificed via carbon dioxide inhalation and cervical dislocation. The entire cecum was immediately excised, the content removed and the tissue cleaned with sterile 0.9% NaCl. The cecal tissue was first cut at the greater curvature and fixed in 10% Formalin solution (Sigma-Aldrich, St. Louis, MO, USA) for histology, then cut longitudinally into two halves and snap-frozen in liquid nitrogen. Cecal tissue and content were stored at −80°C until further processing. All mice handling and dissections were performed in sterile conditions and completed in a biosafety cabinet using sterile instruments. See Fig. 1A for the study design.

### Probiotic strains

The probiotic supplement (Lacidofil), a 95:5 mixture of *Lacticaseibacillus rhamnosus* R0011 (CNCM I-1720) and *Lactobacillus helveticus* R0052 (CNCM I-1722), was provided by Lallemand Health Solutions, Inc. (Montréal, QC, Canada), in lyophilized form and stored at 4°C. The viability of probiotic strains was assessed by plating aliquots of freshly prepared and old (after 48 h) drinking water on De Man, Rogosa, and Sharpe (MRS) agar (Difco Lactobacilli MRS Broth, BD Biosciences, Sparks, MD, USA).

### DNA extraction

Total DNA was extracted from half of the cecal content using the E.Z.N.A Stool DNA Kit (Omega Bio-tek, Inc., Norcross, GA, USA) as previously described (28). DNA concentration and purity were assessed with the NanoDrop 2000 Spectrophotometer (Thermo Fisher Scientific, Foster City, CA, USA). DNA samples were stored at −20°C.

### Quantification of probiotic strains and total bacteria using real-time PCR

Quantification of total bacteria and probiotic strains in the cecal content was assessed in triplicate using the 7900HT Fast Real-Time PCR System and 384-well optical plates (Applied Biosystems, Thermo Fisher Scientific). Total bacteria were quantified using 10 ng/µL of total DNA, TaqMan Gene Expression Master Mix (Applied Biosystems, Thermo Fisher Scientific, Foster City, CA, USA), and custom TaqMan assay targeting the 16S rRNA gene (29) in a final volume of 10 µL per reaction (default thermal profile settings). Total bacterial counts were calculated using a pGEM T-Easy-16S rRNA based standard curve and data expressed as log_10_ 16S copies/g of cecal content.

Probiotic strains were quantified using Power SYBR Green PCR Master Mix (Applied Biosystems, Thermo Fisher Scientific, Foster City, CA, USA), 20–50 ng/μL of total DNA, and strain-specific primers for *L. rhamnosus* R0011, targeting the 113A29 bacteriophage head protein (30) and for *L. helveticus* R0052, targeting the open reading frame 5 of the pIR52-1 plasmid (31), in a final volume of 10 µL per reaction. The thermal cycle profile was modified as follows: 10 min at 95°C, 40 cycles of 15 s at 95°C then 1 min at 60°C. After amplification, a dissociation curve analysis was performed to ensure the specificity of the reaction and verify the absence of primer dimers. The number of cells for each probiotic strain was calculated from culture-based standard curves and expressed as CFU/g of cecal content, normalized to total bacterial counts as previously described (28).

### 16s rRNA gene amplicon sequencing

The sequencing library preparation was performed according to Illumina 16S Metagenomic Sequencing Library Preparation guide (part no. 15044223 Rev. B), with the exception of using Qiagen HotStar Master Mix (Qiagen, Hilden, Germany) for the amplicon PCR. The primers 16S-IlluF (5′-CCTACGGGNGGCWGCAG-3′) and 16S-IlluR (5′-GACTACHVGGGTATCTAATCC-3′) targeting the V3–V4 hypervariable regions were used to amplify a 550 bp fragment (32). The PCR amplification was performed for 25 cycles using an annealing temperature of 55°C. Purified PCR products were then loaded on an Illumina MiSeq and sequenced using the MiSeq 500-cycle V3 Kit (Illumina, San Diego, CA, USA).

### RNA extraction

Total RNA was extracted from cecal tissue and the second half of the cecal content using the *mir*Vana miRNA Isolation Kit (Ambion, Life Technologies, Waltham, MA, USA) as per the manufacturer’s protocol. RNA concentration and purity were assessed with the NanoDrop 2000 Spectrophotometer (Thermo Fisher Scientific, Foster City, CA, USA). RNA samples were stored at −80°C.

### RNA quality control

The quality of the cecum and cecal content total RNA samples was assessed using the Agilent 2100 Bioanalyzer (Agilent Technologies, Inc., Santa Clara, California, USA) and RNA 6000 Pico Kit. The electropherograms were analyzed with the 2100 Expert Software version B.02.08.SI648 (Agilent Technologies, Inc., Santa Clara, CA, USA).

### MicroRNA profiling

Twenty-four pairs of cecum and content RNA samples extracted from 24 male offspring (a total of 48 samples) were used for miRNA profiling with the nCounter Mouse v1.5 miRNA Expression Assay Kit (NanoString Technologies, Inc., Seattle, WA, USA) (miRBase built version 15). The experiments were performed at the Princess Margaret Genomics Center Toronto, Canada (www.pmgenomics.ca), using 100 ng of total RNA according to the manufacturer’s instructions.

For the control group, 12 samples were analyzed: 1 male offspring/dam/PND (*n* = 4 male offspring/PND). For the probiotics group, 12 samples were analyzed across all dams: *n* = 3, *n* = 5, and *n* = 4 male offspring for PND14, PND21, and PND36, respectively.

### Cecal and cecal content microRNA quantification using droplet digital PCR (ddPCR)

PND- and group-associated miR-199a expression was confirmed in male samples used for NanoString and validated using additional non-littermate males (*n* = 6–7 male offspring/PND for control and *n* = 3–5 male offspring/PND for probiotics for each of tissue and content) and littermate females (*n* = 3–4 female offspring/PND for control and *n* = 4–5 female offspring/PND for probiotics for each of tissue and content) by ddPCR. Briefly, 10 ng of total RNA was first reverse-transcribed (RT) using TaqMan MicroRNA Reverse Transcription Kit and microRNA specific primers for miR-199a-5p (Applied Biosystems, Thermo Fisher Scientific, Foster City, CA, USA).

The ddPCR was performed in 20 µL of a reaction mixture containing 10 µL of digital PCR^TM^ Supermix (Bio-Rad, Hercules, CA, USA), 1 µL of TaqMan assay (Applied Biosystems, Thermo Fisher Scientific, Foster City, CA, USA), and 5 µL of RT solution. The mixture was loaded into DG8 cartridges (Bio-Rad Laboratories, Hercules, CA, USA) with 70 µL of Droplet Generation Oil for Probes (Bio-Rad Laboratories, Hercules, CA, USA) and then placed into the QX100 Droplet Generator (Bio-Rad Laboratories, Hercules, CA, USA). The generated droplets were then transferred into a 96-well PCR plate to run the PCR following the manufacturer’s protocol. After run completion, the plate was loaded on a QX100 Droplet Reader (Bio-Rad Laboratories, Hercules, CA, USA) for the quantification of total miRNA copies per reaction. The data were reported as total miRNAs/g of cecal or cecal content as follows:


$$\frac{Adjusted\;miRNA\;copies/reaction}{Weight\;of\;sample\;(g)}=total\;miRNAs/g\;of\;cecal\;content$$

### Histology

Cecal tissue (*n* = 3–4 male and female offspring/PND/group) fixed in 10% Formalin solution was processed, embedded in paraffin, cut into 5-µm-thick sections, and stained with hematoxylin and eosin (H&E) and alcian blue/periodic acid-Schiff (AB/PAS) stains following standard procedures. Parameter scoring was completed by a pathologist blinded to the experimental conditions for the following parameters: normal architecture (yes/no), presence of inflammation (yes/no), goblet cell count/40 × field, and crypt depth (µm). Parameter scoring was performed and replicates of five were recorded. Microscopic images were captured at a magnification of 200 × and visualized at a scale bar of 100 µm.

### Immunofluorescence

Ki67 immunofluorescence staining of cecal tissue sections (*n* = 3–4 male and female offspring/PND/group) was performed as previously described (33). Briefly, tissue sections were incubated overnight at 4°C with 1 in 500 dilutions of primary antibodies (Abcam, Cambridge, UK), then incubated with 1 in 1,000 diluted fluorescent secondary antibodies (Invitrogen, Carlsbad, CA, USA) for 2 h at room temperature. Antibody-labeled cells were counted by a pathologist in a blinded manner (nine replicates/section), and data were expressed as Ki67+ cell count/crypt. Microscopic images were captured at a magnification of 200 × and visualized at a scale bar of 57 µm.

### 16s rRNA gene amplicon sequencing data analysis

Amplicon sequences were analyzed as previously described (34, 35). The pair-end reads were first quality-controlled, then paired-end assembled, and clustered at 97% similarity. The operational taxonomic units (OTUs) were assigned using the Ribosomal Database Project (RDP) classifier (v2.5) with a custom Greengenes (v13_5) training set (34 - 37). Alpha diversity (Shannon index) and beta diversity (Bray–Curtis dissimilarity matrix) were assessed using QIIME 1 software (38).

The linear discriminant analysis (LDA) effect size (LEfSe) method (LDA score >2) was used to identify discriminatory taxa between groups (39). To infer differential relative abundances in bacterial taxa, microbiota data were also analyzed with the negative binomial distribution method (R/Bioconductor DESeq2 package) as in reference (40); an FDR-adjusted *P-*value with a cut-off value of 0.05 was used as a threshold.

### MicroRNA profiling data analysis

Cecum miRNA data were normalized with NanoStringNorm v1.2.0 (41) by applying a technical assay variation adjustment (using geometric mean counts across all probes as the normalization factors), a background (mean of negative probes counts) correction, and a sample content variation adjustment (using the total sum of all the endogenous probes) before final log_2_ transformation. Multidimensional scaling (MDS) was performed to show samples similarity/ dissimilarity. Multiple linear regression analysis was then performed, in which the expression of miRNAs was modeled as a linear function of three predictors: PND, group, and dam, where PND was treated as a nominal variable and group and dams were treated as categorical variables. The resulting model was subjected to ANOVA to assess the statistical significance of individual predictors. The overall goodness-of-fit was assessed by *F* test. The *P-*values were adjusted for multiple testing by applying the false discovery rate (FDR) method. Three subsets of miRNAs were created, those significantly (FDR-adjusted *P*-value < 0.01) associated with PND, with group, and with both PND and group, while the goodness-of-fit FDR was <0.01.

Cecal content miRNA data analysis was performed in a similar manner but without adjusting for technical assay variation; PND-associated miRNAs were identified by FDR-adjusted *P*-value < 0.15, and PND- and group-associated miRNAs were identified by FDR-adjusted *P*-value < 0.25 while applying the model goodness-of-fit significance as an additional criterion.

For PND- and group-associated miRNAs subsets, heatmaps showing unsupervised hierarchical clustering (using Euclidean distance) of significantly changed miRNAs were created (pheatmap v1.0.8). Panel plots were generated to visualize the expression of the miRNAs across PNDs and groups interactions. All the analyses were performed in R v3.3.2 (42).

### Cecal microRNA-gene targets, Gene Ontology enrichment analysis, and regulatory network

The predicted miRNA-gene targets of the PND-associated, group-associated, and PND- and group-associated miRNAs were identified using miRWalk2.0 (43, 44). Gene targets supported by less than 9 of the 10 sources and targeted by less than 3 miRNAs (only for PND-associated and group-associated miRNAs) were removed. The gene targets were subjected to enrichment analysis across Gene Ontology biological processes, molecular functions, and cellular components, using the clusterProfiler package (v3.2.14) (45) (BH-adjusted *P*-value < 0.01); annotations were sorted by smallest to largest *q* value, then by largest to smallest gene count to reveal the top 10 annotations. The PND- and group-associated miRNAs, gene targets, and enriched ontologies were then connected to create a network that was visualized and analyzed using NAViGaTOR 3.0 (24). The final network was exported in SVG into Adobe Illustrator to finalize the legends and saved in 300 DPI PNG file format.

### Cecum and cecum content microRNA correlations

NanoString log_2_ normalized read counts or log_2_ miRNA copies (ddPCR data) were used for Pearson correlations using multiple testing-adjusted *P-*values (FDR < 0.1).

### Cecal and cecal content microRNA and cecal content microbiota correlations

Pairwise correlations between cecal miRNAs (log_2_ normalized read counts) and cecal content microbial taxa (relative abundance at L2–L6) and between cecal content miRNAs and cecal content microbial taxa (relative abundance at L2–L6) were assessed using the Kendall and Spearman formulas (significance at *P*-value < 0.05).

Significant associations identified (*T* ≥ 0.44, *P*-value < 0.05) were used to generate correlation networks. The correlation heatmaps were generated using ClustVis (46)(47).

## Data Availability

The raw 16S rRNA gene amplicon sequences were deposited in NCBI Sequence Read Archive (SRA) database under the accession number PRJNA670240. The raw miRNA reads were deposited in NCBI Gene Expression Omnibus (GEO) database under the accession number GSE149418

## SUPPLEMENTAL MATERIAL

Supplemental Figures msystems.00431-23-s0001.pdf Supplemental figures, related to Figures 1, 2, 3, and 4 in the text.

Supplemental Legends msystems.00431-23-s0002.docx Legends describing the supplemental figures and tables.

Table S1 msystems.00431-23-s0003.xlsx Complete list of cecal and cecal content miRNAs and Pearson correlations.

Table S2 msystems.00431-23-s0004.xlsx miRNA-gene targets for PND-, group-, and PND x group-associated miRNAs.

Table S3 msystems.00431-23-s0005.xlsx Enriched Gene Ontology (GO) annotations related to the PND-, group-, and PND x group-associated miRNAs.

Table S4 msystems.00431-23-s0006.xlsx miRNAs differently expressed in the probiotic group at earlier time points compared to the ones in the control group at later time points.

Table S5 msystems.00431-23-s0007.xlsx miRNA-microbiota Kendall correlations in cecal and cecal contents.

ASM does not own the copyrights to Supplemental Material that may be linked to, or accessed through, an article. The authors have granted ASM a non-exclusive, world-wide license to publish the Supplemental Material files. Please contact the corresponding author directly for reuse.
