# Supplementary material for: Intestinal microRNAs and bacterial taxa in juvenile mice are associated, modifiable by allochthonous lactobacilli, and affect postnatal maturation
Source: mSystems. 2023 Jul 18;8(4):e00431-23. doi: 10.1128/msystems.00431-23 (PMC10469672; doi:10.1128/msystems.00431-23)
Supplement: Supplemental Figures — , related to Figures 1, 2, 3, and 4 in the text. [file msystems.00431-23-s0001.pdf]

Supplemental Figures

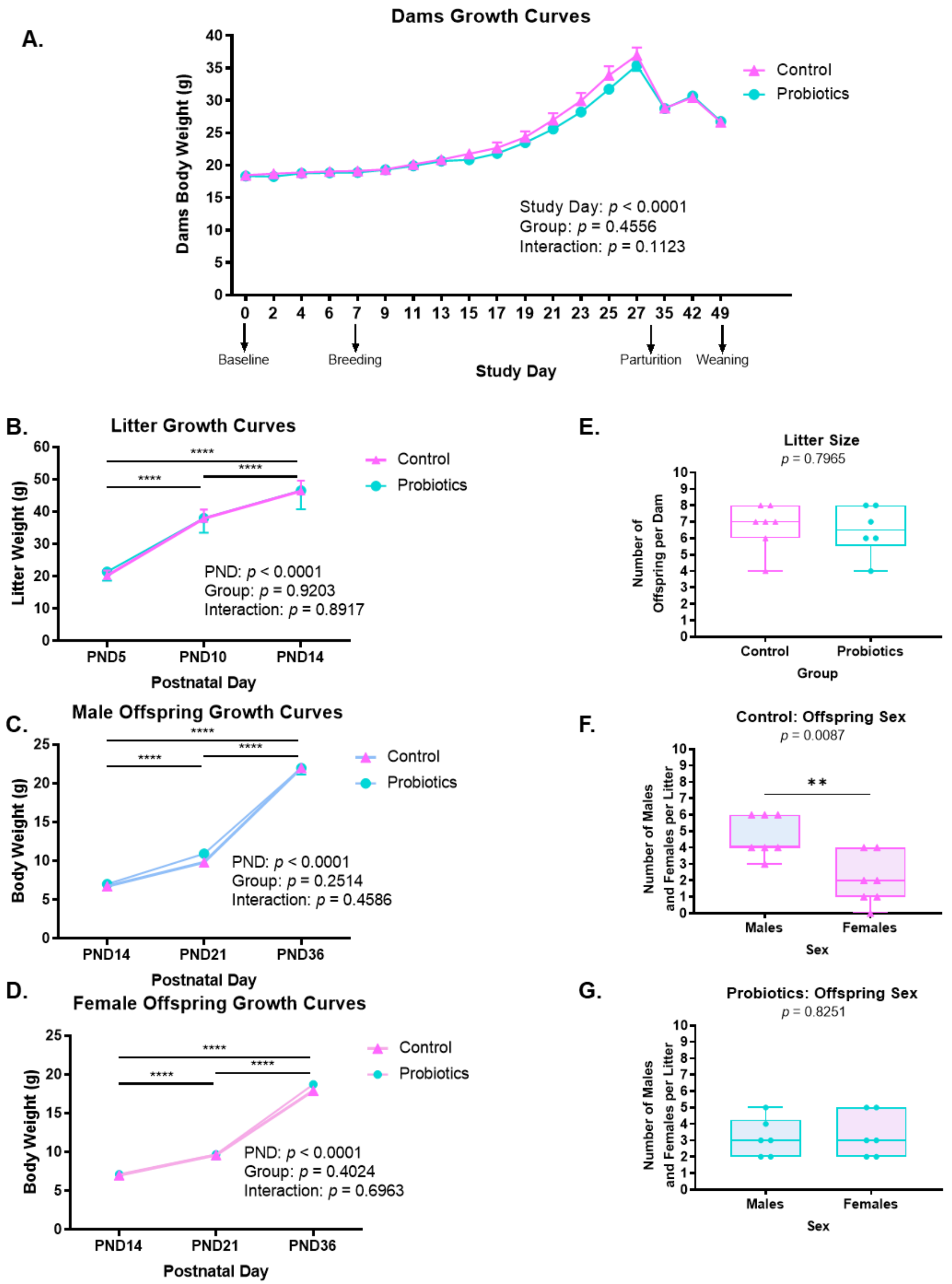

Figure S1. Probiotics have no Effect on Dam and Litter Characteristics. Related to Figure 1.

(A) Dams' body weight throughout the study (Mean ± SEM; n = 6-7 dams/group; Two-way mixed ANOVA and Bonferroni- adjusted p-value).

(B) Litter weight (Mean ± SEM; n = 6-7 litters/group, Two-way mixed ANOVA and Bonferroni-adjusted *p-value*; **** $p < 0.0001$).

(C-D) Male offspring (C) and female offspring (D) body weight growth curves (Mean ± SEM; n = 4-13 offspring/sex/PND/group and; Two-way ANOVA and Bonferroni- adjusted *p-value*; **** $p < 0.0001$).

(E) Litter size (Mean ± SEM; n = 6-7 litters/group; unpaired t-test with Welch's correction).

(F-G) Offspring sex for the control group (F) and probiotics group (G) (Mean ± SEM; n = 6-7 litters/group; Mann-Whitney U test, ** $p < 0.01$).

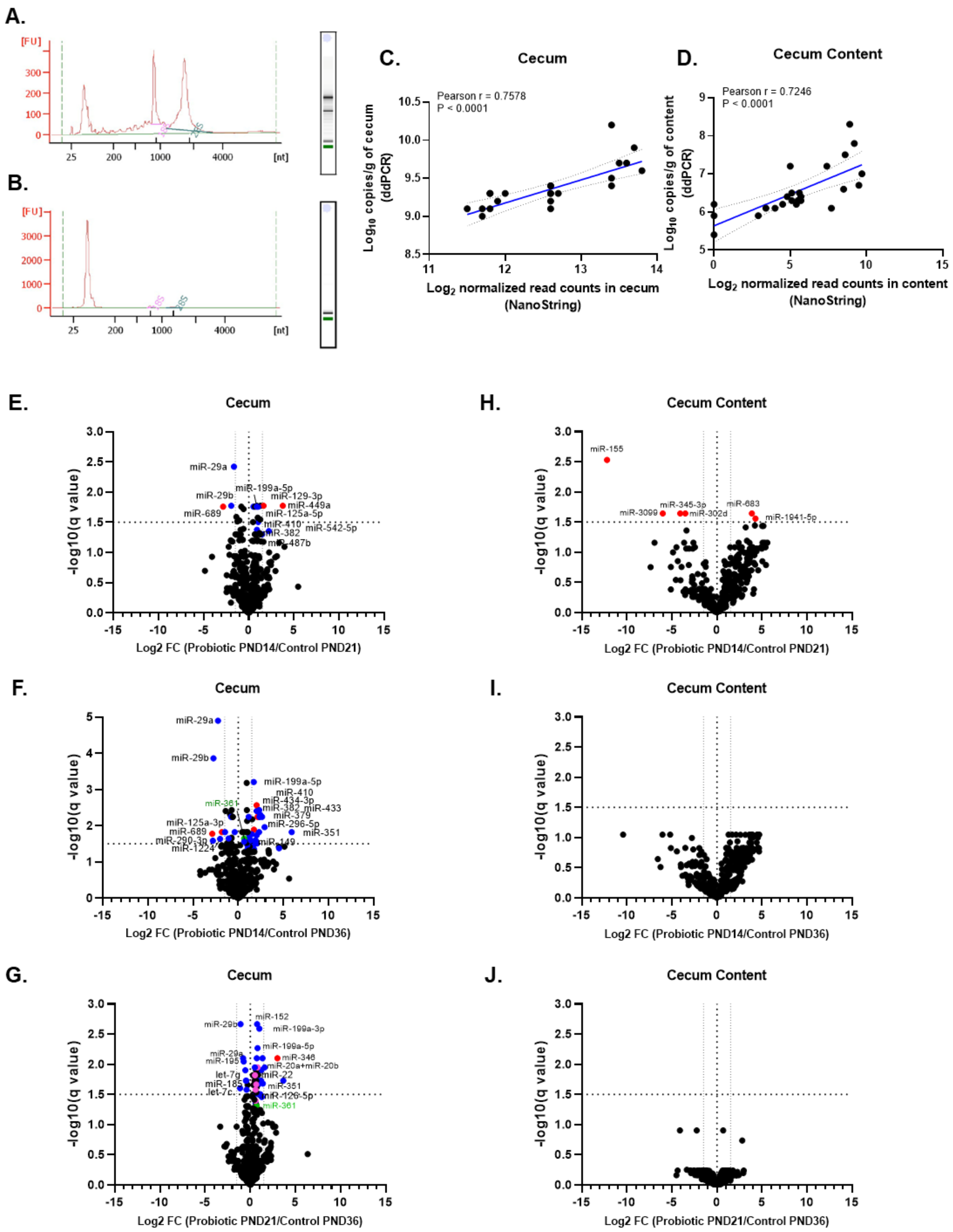

Figure S2. RNA Quality Control and miRNA expression analyses. Related to Figures 1 and 2

(A-B) Representative electropherograms of cecal (A) and cecal content (B) depicting 18S and 28S peaks for cecum only.

(C-D) Pearson correlations showing the significant associations between $\log_2$ normalized miR-199a-5p counts (NanoString) and $\log_2$ copies (ddPCR) per g of cecum (C) and cecum content (D). *p-value* < 0.0001

(E-J) Volcano plot of $\log_2$ fold change (FC) of miRNAs in probiotic groups at earlier time-points compared to later time-points in the control group. In cecum (E, F, G) and cecum content (H, I, J). Each dot represents one miRNA. The Blue, purple and green colors indicate PND-, Group- and PND*Groups-associated miRNAs, respectively. The red color shows significantly differently expressed miRNAs (not previously identified in figures 1 and 2). (Unpaired t-tests. FDR-adjusted *p-value* < 0.05).

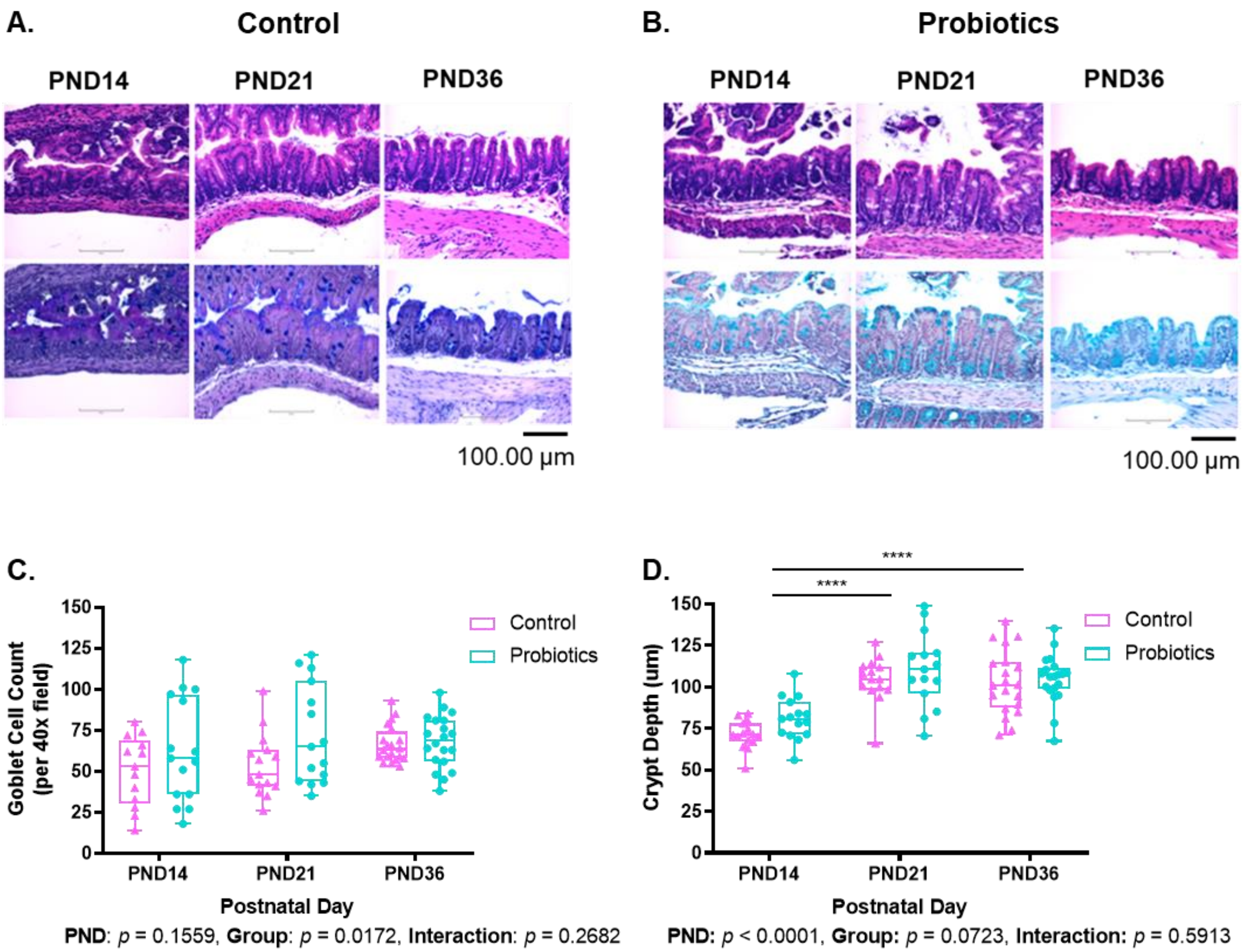

Figure S3. Epithelial Cell Proliferation Analysis. Related to Figure 2

(A-B) Representative H&E and AB/PAS staining images depicting cecal tissue architecture in control (A) and probiotics (B) group at PND14, PND21, and PND36. See Figure 2F.

(C-D) Mean ± SEM of goblet cell count/40× field (C) and crypt depth (D) (n = 5 replicates for 3-4 offspring/PND/group; Two-way ANOVA and Bonferroni-adjusted *p-value*; **** $p < 0.0001$). See Figure 2G.

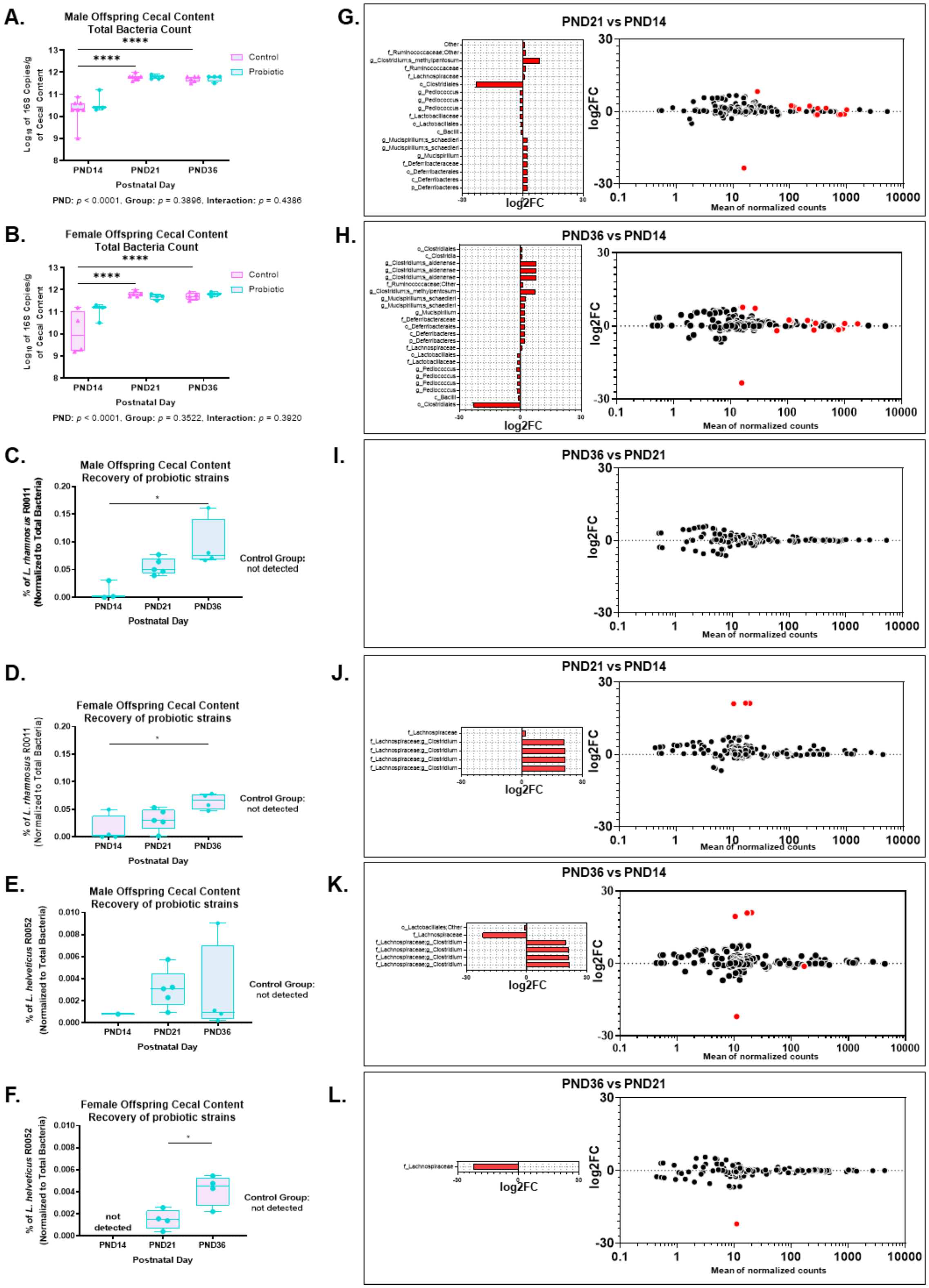

Figure S4. Probiotics Affect the Developing Gut Microbiota. Related to Figure 3

(A-B) Mean ± SEM of $\log_{10}$ CFU/g of cecal content of total bacteria for male offspring (*n* = 3-7 male offspring/PND/group (A), and female offspring (*n* = 3-5 female offspring/PND/group) (B) (Two-way ANOVA and Bonferroni-adjusted *p-value*; ***$p < 0.001$, **** $p < 0.0001$).

(C-D) Mean ± SEM of *L. rhamnosus* R0011 as a percentage of total bacteria for male offspring (*n* = 3-5 male offspring/PND/group) (C), and female offspring (*n* = 4-5 female offspring/PND/group) (D). Each dot represents individual offspring (Kruskal-Wallis test and Dunn's-adjusted *p-value*; * $p < 0.05$, ***$p < 0.001$). The strain was not detected in control samples.

(E-F) Mean ± SEM of *L. helveticus* R0052 as a percentage of total bacteria for male offspring (*n* = 1 male offspring for PND14 and *n* = 4-5 male offspring for PND21 and PND36 for probiotics) (E), and female offspring (*n* = 0 female offspring for PND14 and *n* = 4 female offspring for PND21 and PND36 for probiotics) (F). Each dot represents individual offspring (Female offspring in panel F were analyzed using unpaired *t*-test with Welch's correction; * $p < 0.05$. Male offspring in panel E were analyzed using Kruskal-Wallis test). Female offspring at PND14 and control samples were not detected for this strain.

(G-H-I-J-K-L) Pairwise comparison using DeSeq2 analysis between the developmental ages in the control (G-H-I) and probiotic (J-K-L) groups.

The right panels show the MA-plots of the log2 fold changes over the mean of normalized OTU counts. Red refers to the significantly differently abundant OTUs (Adjusted *p*-value < 0.005). Left panels show the log2 fold changes of OTUs identified in the MA-plots as significantly changing at PND21 vs PND14, PND36 vs PND14, and PND36 vs PND21 in the control and probiotic groups.

A. B. C.

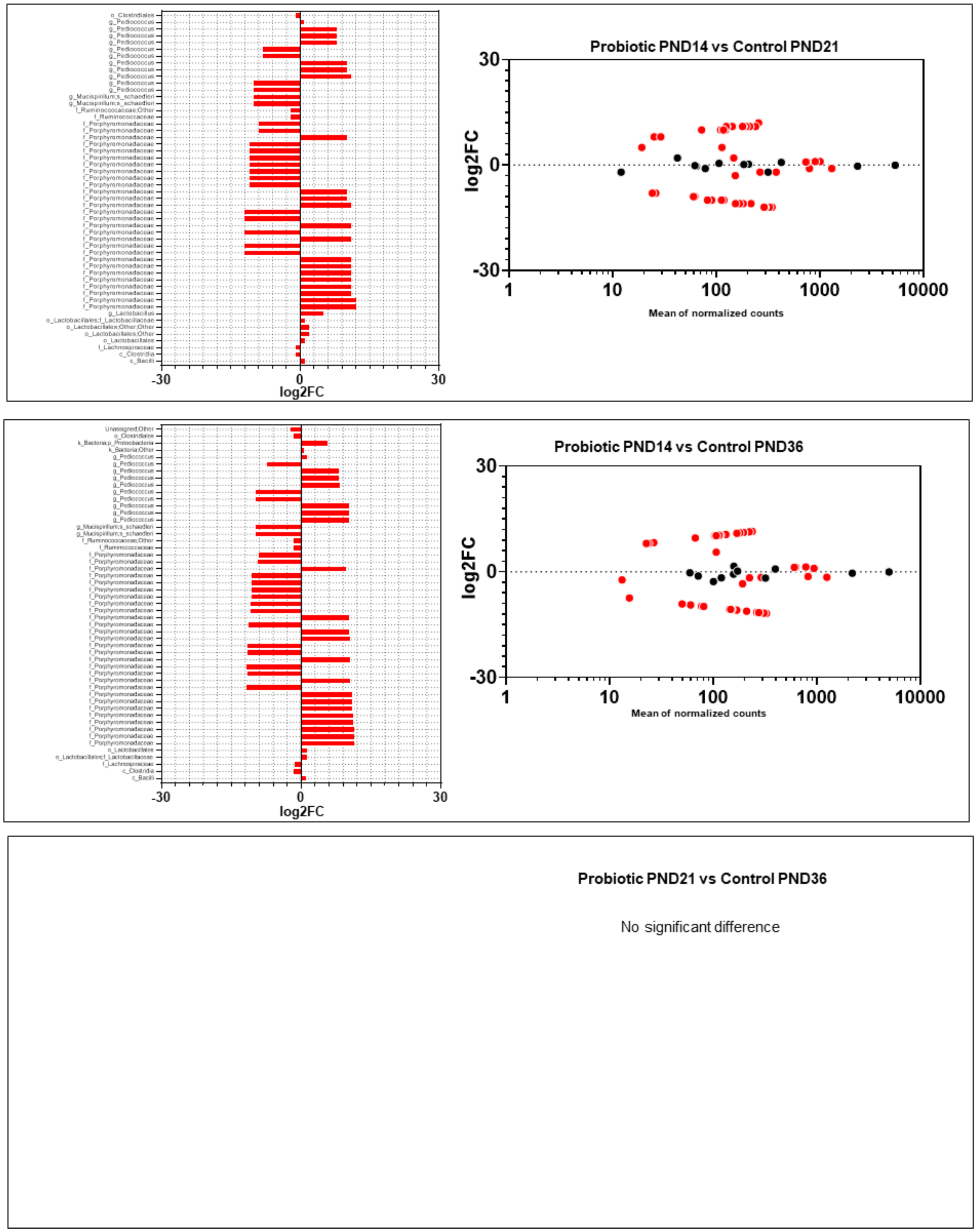

Figure S5. Probiotics contribute to the maturation of the microbiota. Related to Figure 3 and Figure S6

(A-C) Differential relative abundance analysis of the microbiota composition between probiotic treated pups at earlier time-points and that of the control group at later time-points, using DESeq2

functions. The right panels show the MA-plots of the log2 fold changes over the mean of normalized OTU counts. Red dots indicate the significantly differently abundant OTUs (Adjusted *p-value* < 0.005). Left panels show the log2 fold changes of OTUs identified in the MA-plots as significantly changed in probiotic group compared to the control group at PND14 (probiotic) vs PND21 (control) (A) and PND21 (probiotic) vs PND36 (control) (B)

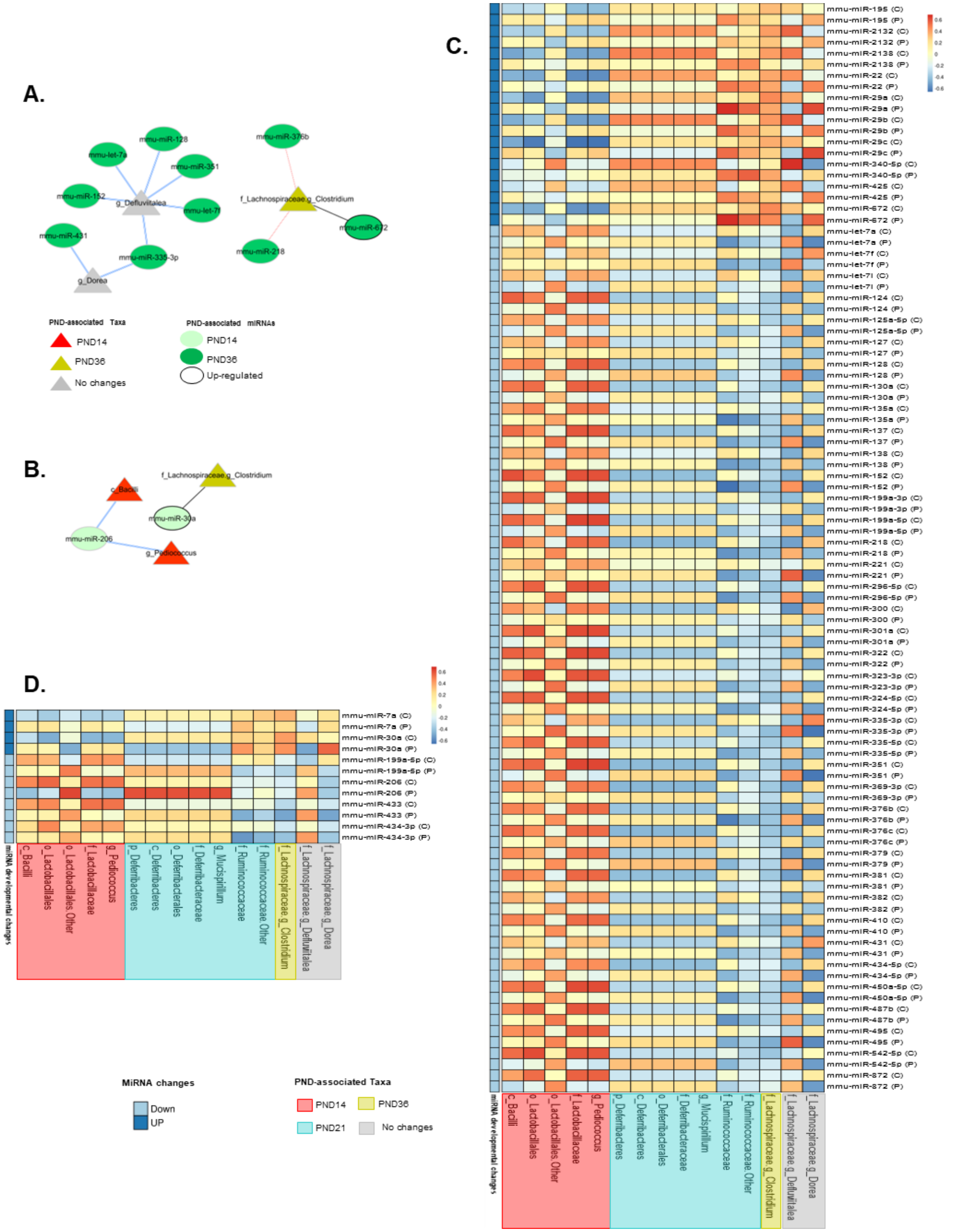

Figure S6. Probiotics Affect the MiRNA-Microbiota Relationship. Related to Figure 4

(A-B) MiRNA-taxon correlation pairs in the cecum (A) and the content (B), showing a site-specific association miRNA-bacteria. Nodes colors refer to the age-associated miRNA and/or taxa

significant changes. Red edges indicate negative correlation (in both groups), black edges indicate positive correlation (in both groups), while the blue parallel lines refer to correlations antagonized by probiotics. The association networks were visualized using Cytoscape 3.7.1 (49) (C-D) Heatmaps showing the correlations that are disrupted by probiotics in cecum (C) and content (D), with miRNAs in rows (Control (C) and Probiotic (P) groups) and bacterial taxa in columns. The orange color in the heatmap indicates positive and the blue color indicates negative correlation ($T \geq 0.44$, p-value <0.05).
